# A Randomization-Based, Model-Free Approach to Functional Neuroimaging: A Proof of Concept

**DOI:** 10.3390/e26090751

**Published:** 2024-09-02

**Authors:** Matan Mazor, Roy Mukamel

**Affiliations:** 1 All Souls College, University of Oxford, Oxford OX1 4AL, UK; 2 School of Psychological Sciences, University of Oxford, Oxford OX1 2JD, UK; 3 Sagol School of Neuroscience, Tel-Aviv University, Tel Aviv 69978, Israel; rmukamel@tau.ac.il; 4 School of Psychological Sciences, Tel-Aviv University, Tel Aviv 69978, Israel

**Keywords:** fMRI, model-free analysis, randomization

## Abstract

Functional neuroimaging analysis takes noisy multidimensional measurements as input and produces statistical inferences regarding the functional properties of brain regions as output. Such inferences are most commonly model-based, in that they assume a model of how neural activity translates to the measured signal (blood oxygenation level-dependent signal in the case of functional MRI). The use of models increases statistical sensitivity and makes it possible to ask fine-grained theoretical questions. However, this comes at the cost of making theoretical assumptions about the underlying data-generating process. An advantage of model-free approaches is that they can be used in cases where model assumptions are known not to hold. To this end, we introduce a randomization-based, model-free approach to functional neuroimaging. TWISTER randomization makes it possible to infer functional selectivity from correlations between experimental runs. We provide a proof of concept in the form of a visuomotor mapping experiment and discuss the possible strengths and limitations of this new approach in light of our empirical results.

## 1. Introduction

fMRI relies on the coupling between blood flow and neural firing to infer neural activity from the blood oxygenation level-dependent (BOLD) signal—a signal that changes in proportion to the ratio of oxy- to deoxyhaemoglobin in the blood [1]. This signal is correlated with both neural firing and field potentials [2,3,4]. While much progress has been made in understanding the mechanisms that underlie this relation (see [5], for a review), it is still far from being completely understood, with ongoing research into factors such as the specific roles of glial cells and the electrophysiological basis of negative BOLD signals [5,6].

The predominant approach to fMRI signal modelling treats the measured fMRI signal as the convolution of neural activity with a Hemodynamic Response Function (HRF), accompanied by Gaussian noise [7]. In the past two decades, this linear transform model has proven very useful. Its simplifying assumptions allow for the use of the General Linear Model (GLM), which is employed in mass univariate Statistical Parametric Mapping (SPM, [8]) and, often, as a preliminary step to multivariate approaches such as Multi Voxel Pattern Analysis (MVPA, [9]) and Representational Similarity Analysis (RSA, [10]).

The assumption of linearity of the BOLD signal comprises three assumptions regarding the measured signal [7], namely that it is additive (*additivity*), proportional to stimulus salience (*scaling*), and consistent across time points (*shift invariance*). In addition, it is tacitly assumed that the neurovascular coupling mechanism is consistent across brain regions, allowing for the convolution of experimental events with the same canonical HRF throughout the brain.

These assumptions were shown to be reasonable with respect to the human primary visual area [11]. However, later work revealed violations of the principles of additivity [12,13,14] and scaling [14,15]. The HRF was also shown to vary between different brain regions [16,17,18,19] and cortical layers [20] and, to a lesser degree, across time within the same subject [21]. The HRF also varies between populations [22], such as the elderly [23,24], children [25], and clinical patients [26,27,28]. Variation is not limited to the latency, duration, or magnitude of the hemodynamic response function but is also evident in its shape and sign. Taylor, Kim, and Ress (2018) reported consistently negative HRFs in more than 25% of the cortex [18], and Pucket, Mathis, and Deyoe (2014) reported such inverted HRFs for *positive* neural activations, even in visual cortices, which are assumed to be well captured by the linear transform model [19]. Unmodelled variability in the HRF limits the sensitivity of fMRI model-based analyses, especially when using event-related experimental designs and analyses, which are more sensitive to modelling assumptions.

Improving the precision of the model is one solution to this problem. This can be done by allowing more freedom in describing the HRF shape [6,29] and modelling the deviations from linearity [30]. However, a caveat of this approach is that it introduces complications to the model, compromising statistical power and making results harder to interpret [30]. An alternative solution is to use model-free approaches, bypassing the need to commit to a specific model. Examples of model-free applications in fMRI analysis include the use of within-subject, inter-voxel correlation as an index of functional connectivity [31,32]; the use of within-subject, within-voxel correlation for signal reliability assessment across different time points [33]; and the use of inter-subject, within-voxel correlations to infer commonalities and interactions in brain processing across subjects [34,35,36].

The above approaches estimate the correlations between empirical time-series data to learn about brain function. In doing so, they rely on the fact that time series are locked to the same series of events, whether external [33,34,35,36] or internal [31], so that any common activity can be ascribed to the stimulus or to the connectivity of the network. Here, we introduce a new member to this family, namely a model-free, correlation-based approach that measures the difference in the correlations of BOLD responses to *different series of external events* (see Table 1). Correlations are computed within subjects and within voxels but across conditions. Our method comprises two steps. First, the experiment is designed such that experimental runs are consistent with respect to certain experimental dimensions and are inconsistent with respect to others (TWISTER design; see Section 2.1). Secondly, analysis is performed in a model-free manner, relying on differences in temporal correlations between experimental runs as a measure of functional selectivity (Temporal Consistency Asymmetry (TCA); see Section 2.2).

## 2. Materials and Methods

We describe a two-step approach to functional brain imaging that does not assume a specific generative model of the measured signal. Our approach comprises an experimental design that manipulates inter-run consistency along specific dimensions of interest (TWISTER, Section 2.1) and a quantitative measure of the asymmetry of inter-run consistency in the BOLD response (TCA, Section 2.2).

### 2.1. TWISTER

**Figure 1:**
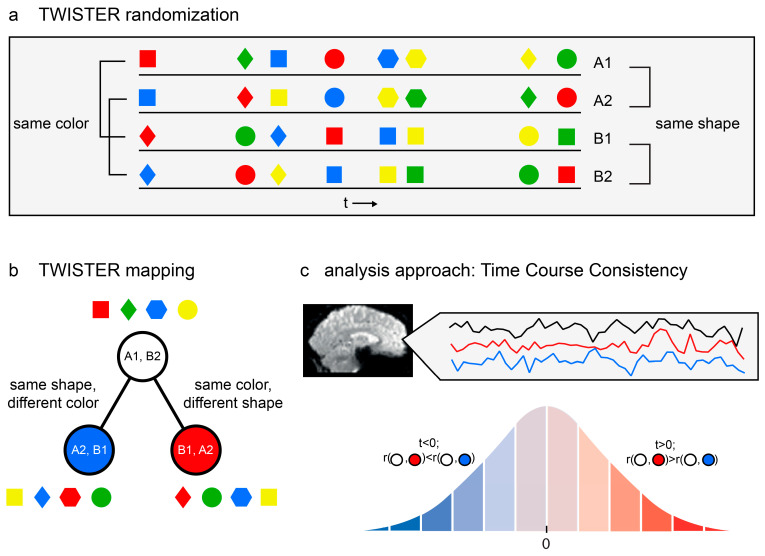
Model-free functional brain analysis. (**a**) An example of TWISTER randomization, manipulating colour and shape as the dimensions of interest. The four lines stand for the four experimental runs. Notice that pairs A1, A2 and B1, B2 are consistent with respect to shape but twisted along the colour dimension, whereas A1, B1 and A2, B2 are consistent with respect to colour but are twisted along the shape dimension. (**b**) TWISTER mapping for the said experiment. Each circle represents the temporal concatenation of two experimental runs into one long time series. (**c**) Temporal Consistency Asymmetry (TCA) is computed for a given voxel with respect to three activation time series. The analysis is designed to examine whether the seed time series is more consistent with the red or blue reference time series. Using the Hotelling–Williams test and based on the correlation between the three time series, a *t* value is computed. The *t* value is then compared with the appropriate cumulative distribution function to extract a *p* value. A statistical parametric map is then constructed using voxel-based or cluster-based thresholding, where blue voxels indicate stronger temporal consistency along the blue dimension, and the opposite is true for red voxels.

Unlike traditional experimental designs that are optimized for identifying evoked responses to particular *conditions* or *event types* (for example, see [37]), the TWISTER experimental design is optimized for identifying brain regions that are more sensitive to certain *dimensions* along the stimulus space than to other dimensions. A TWISTER experiment comprises four experimental runs of equal duration (A1, B1, A2, and B2). The experiment is created in two steps. First, run A1 is formed by randomly dispersing a fixed number of events along a temporal interval of a predefined duration. This step is executed separately for each participant to control for order effects and to increase generalizability. Secondly, the three remaining runs are created by taking run A1 as a reference, keeping the timing of events identical, and twisting (that is, randomizing or inverting) the events along the first dimension (B1), the second dimension (A2), or both (B2; see Figure 1). The resulting four runs can be used in random order. Thus, when projected onto a space spanned by the two dimensions of interest, run A1 is aligned with run A2 along the first dimension and with run B1 along the second dimension, and the opposite is true for B2.

Consequently, voxels can be projected onto the same two-dimensional space according to the temporal consistency along the two twisted stimulus dimensions, as reflected in the correlation coefficients between time series. A brain region whose time series during run A1 is more consistent (i.e., has a higher correlation) with its activation pattern during run A2 than during run B1 is more sensitive to the first dimension (A vs. B) than to the second dimension (1 vs. 2). Similarly, a brain region whose time series during run B2 is more consistent with its activation pattern during run A2 than during run B1 is more sensitive to the second dimension (1 vs. 2) and less to the first (A vs. B).

### 2.2. Temporal Consistency Asymmetry (TCA)

TWISTER randomization aligns the asymmetry in the voxel activation space (‘the BOLD time-series response of voxel x during run A1 is more consistent, that is, more temporally correlated, with its response during run B1 than with its response during run A2’) with asymmetry in the voxel’s representation space (‘voxel x is more sensitive to the second experimental dimension (e.g., colour) than to the first (e.g., shape)’). To quantify the temporal consistency asymmetry (TCA) for each voxel, we propose the following procedure:

1. Extract temporal consistency measures.
  For every voxel,
  - Set the *seed time series* to be the voxel’s time series in a given run (for example, A1). Set the *red reference* to be the time series of the same voxel in a different run that is consistent along one dimension but inconsistent along the second dimension (for example, A2). Set the *blue reference* to be the time series of the same voxel in a different run, consistent only along the second dimension (B1). This step can be performed using the concatenation of two or more standardized time series, for example, setting the concatenation of [A1, B2] as the seed time series and the concatenation of [A2, B1] and [B1, A2] as red and blue references, respectively.
  - Compute Pearson’s correlation coefficient between each pair of time series (seed and red reference—*r_sr_*; seed and blue reference—*r_sb_*; red and blue references—*r_rb_*).
  - Set any negative correlation to 0.
2. Extract voxel-wise estimates for the number of independent time points.

To account for temporal autocorrelations in the fMRI time series, compute the voxel-wise effective sample size (*ESS*) for each of the three time series using Neal’s approximation [38].

$$ESS=N/(1+2\sum_{k=1}^{\infty }ACF\left(k\right))$$

where *N* is the actual number of acquired time points and *ACF* is the autocorrelation function for a temporal delay (*k*). The resulting number is an estimate of the number of truly independent time points in the time series. To obtain a robust estimate for the voxel’s *ESS*, average the three *ESS* estimates (one for each run) and apply robust spatial smoothing [39], using *ESS* estimates of neighbouring voxels to reduce random variation.

3. Extract voxel-wise temporal consistency asymmetry (TCA) measures.
  For every voxel,
  - Use the Hotelling-Williams test [40,41]—a test for comparing the strength of two dependent correlations [42]—to test the null hypothesis that the seed’s temporal consistency with the red reference is equal to its temporal consistency with the blue reference, i.e., *r_sr_* = *r_sb_*, using the following formula:
    $$T=\left(r_{sr}-r_{sb}\right)\sqrt{\frac{\left(ESS-1\right)\left(1+r_{rb}\right)}{2(\frac{\left(ESS-1\right)}{\left(ESS-3\right)}\left|R\right|+{\underline{r}}^{2}{\left(1-r_{br}\right)}^{3}}}$$
    where |R| is the determinant of the 3 × 3 correlation matrix containing the coefficients being tested. This results in a *t* value. Positive *t* values correspond to *r_sr_* > *r_sb_* (the seed time series is more consistent with the red than the blue reference). Similarly, negative values indicate that *r_sr_* < *r_sb_* (the seed time series is more consistent with the blue than with the red reference).
  - To generate a voxel-wise *p* value, compare the *t* statistic against a *t* distribution with df = *ESS* − 3.

The resulting *t* map can be propagated to a group-level analysis (for example, in an ordinary least squares procedure) or thresholded and visualized at the individual subject level.

Step 1.c is included to avoid the interpretation of negative correlations between time series of the same voxel. While a positive correlation suggests higher consistency than zero correlation, it is unclear how one should interpret negative correlations in the context of temporal consistency. As an example, consider a voxel whose correlation along the red dimension (*r_sr_*) equals −0.6, while its correlation along the blue dimension (*r_sb_*) equals 0. Given an effective sample size of 100 and a zero correlation between the red and blue references, the test results in T(97) = −5.0, a significant result in favour of stronger consistency along the blue dimension. But this is unwarranted, as the consistency along the blue dimension is null. By setting all negative correlations to 0, we restrict our analysis only to voxels in which the difference between the two correlation coefficients is driven by a positive correlation.

A Matlab implementation of this analysis scheme is available, together with the anonymized and pre-processed functional data, at www.github.com/matanmazor/TWISTER.

## 3. Proof of Concept: Visuomotor Mapping

As a proof of concept, we used TWISTER randomization in a visuomotor task to identify visual and motor regions in a model-free manner.

### 3.1. Participant

One female, right-handed participant, aged 26 at the time of testing, took part in the experiment. The participant had corrected-to-normal vision and no reported history of neurological or psychiatric disease. She provided written informed consent to participate in the study prior to participation and was compensated for her time. The study protocol was approved by the Helsinki committee at Sheba Medical Center and the ethics committee of Tel-Aviv University (Institutional Review Board approval 2026-15).

### 3.2. fMRI Task

The participant lay supine on the scanner bed and viewed visual stimuli back-projected onto a screen through a mirror. Foam pads were used to minimize head motion. We used Matlab (Mathworks) and Psychtoolbox [43] for stimulus presentation. The subject’s eye movements were monitored using an EyeLink 1000 Plus eye tracker.

**Figure 2:**
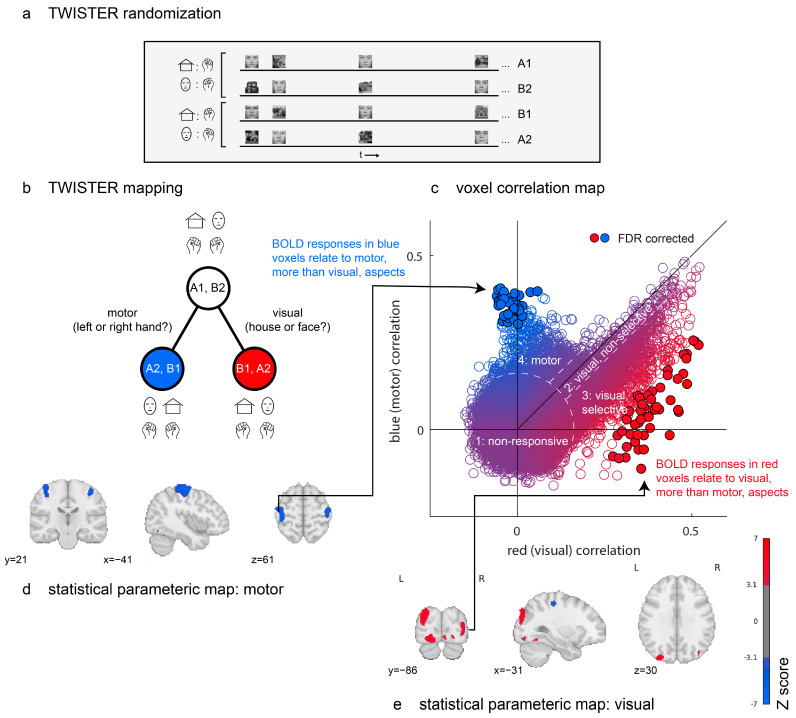
Proof of concept. Design and results from a TWISTER experiment comparing activation selectivity for motor versus visual aspects of an experiment. (**a**) TWISTER randomization: In different experimental runs, the participant responded to a house with a right hand movement and to a face with a left hand movement (runs A1 and B2) or vice-versa (runs B1 and A2). The actual experiment included two sets of four runs, for a total of eight experimental runs. (**b**) TWISTER mapping: Relative to the time course, [A1, B2] (seed), [A2, B1] (blue) preserve motor aspects, while [B1, A2] (red) preserves visual aspects. (**c**) A comparison of correlations of the seed time series with the red versus with the blue reference time series. Individual markers represent voxels, with colour indicating their corresponding *t* values. Filled circles with black outlines survive a correction of the false discovery rate (q < 0.05). Dashed lines separate the voxels into the following four groups: a first group of non-responsive voxels; a second group of responsive but non-selective voxels; a third group of visual, selective voxels; and a fourth group of motor voxels. (**d**,**e**) Uncorrected statistical parametric maps thresholded at *p* < 0.001. Blue clusters are more sensitive to motor aspects of the task and red clusters to visual aspects.

The experiment consisted of eight 4:30 min long experimental runs. During each run, 120 grayscale images appeared on the screen for 500 msec at random times, with the only constraint that event onsets must be separated by at least 500 msec. A total of 60 of the images were face images, and 60 were images of houses, all obtained from publicly available online datasets and cropped to a square format (see Figure 2a). In half of the runs, the participant was instructed to close and open her right hand in response to face images and her left hand in response to house images. In the other half of runs, the instructions were reversed. We used the TWISTER design (see Section 2.2) to manipulate the inter-run consistency along two dimensions of interest: visual category (house or face) and action (left or right hand movement).

### 3.3. MRI Data Acquisition

A Siemens 3-T Prisma scanner (located at the Edersheim-Levi Gitter Center for human brain imaging, Tel Aviv University, Israel) with a 64-channel Siemens Matrix head coil was used to collect all functional and anatomical scans. A single high-resolution structural scan was acquired using a magnetization-prepared rapid acquisition gradient echo (MP-RAGE) sequence (1 × 1 × 1 mm voxels). All functional runs were acquired parallel to the anterior–posterior commissure plane using an echo-planar pulse sequence (38 contiguous interleaved axial slices, 3.5 mm thickness, no gap; TR = 2000 msec; flip angle = 90; TE = 30 msec; in-plane resolution = 3.5 × 3.5 mm; matrix size = 64 × 60).

### 3.4. Image Preprocessing

The acquired data were preprocessed using FEAT v6.00 (FMRI Expert Analysis Tool), a part of FSL (FMRIB software library, version 5.0, www.fmrib.ox.ac.uk/fsl, [44]), accessed on 1 July 2024. Prior to all preprocessing steps, the first two volumes of each run were deleted. Images were then realigned to the central volume of run number 5 to correct for head movements within and across runs and spatially smoothed using a 2.5 mm kernel. The data were then temporally filtered using both a high-pass filter with a cutoff of 50 s and the FILM prewhitening tool. Functional images were registered to the brain-extracted T1 image using boundary-based registration. The anatomical image was registered to the standard MNI space (MNI152, 2 mm) by first performing a linear registration with 12 degrees of freedom, then using the FNIRT nonlinear registration tool with a warp resolution of 10 mm on the linearly registered image.

### 3.5. Results

We applied TCA to three concatenated sets of time series from eight experimental runs. The first “seed” set comprised four runs in which the instruction was to respond with a right hand movement to house images and respond with a left hand movement to face images. The second “red” set comprised four runs in which stimulus categories were aligned with the seed set, but the instructions were reversed; faces were now mapped to a right hand movement and houses to a left hand movement. Finally, the third “blue” set comprised four runs in which stimulus categories were inverted relative to the seed set, as were the instructions. For this reason, left hand movements in the seed set (cued by face images) corresponded to left hand movements in the blue set (cued by house images).

In Figure 2c, we plot the distribution of time-series correlation values for all brain voxels. The resulting two-dimensional space can be broadly separated into four distinct sets of voxels. First, the seed time series of many voxels did not reliably correlate with either red (visual) or blue (motor) time series, resulting in a two-dimensional isotropic gaussian centred at the origin. A second group of voxels follows the diagonal, with positive correlations with both blue and red time series. This group is composed almost exclusively of voxels in the early visual cortex, which activate in response to the mere presence of visual stimuli, irrespective of their visual category. A third group extends below the main diagonal, producing a purple-to-red gradient. Like the second group, these are visual voxels with different levels of content sensitivity. The red dots at the end of this gradient respond reliably differently to face and house images (49 such voxels, marked with black outlines, survive a false discovery rate (FDR) correction for their TCA scores [45]). Finally, a fourth group of voxels shows reliable correlations between the seed and blue (motor) time series but not between the seed and red (visual) time series. These are located in the bilateral primary motor cortex (43 such voxels survive FDR correction).

Together, TWISTER randomization revealed brain regions that respond selectively to certain visual categories or that activate selectively for certain motor actions. This was achieved in a fully model-free manner, using the correlation between runs and no assumptions regarding the mapping from neural activation to hemodynamic responses or about the sequential effects of previous trials on perception and decision making. Critically, the brain maps presented in Figure 2d,e were generated without any design matrix.

## 4. Discussion

We provide a first proof of concept for a novel approach to fMRI experimental design and analysis. This new scheme is unique in two ways. First, unlike the commonly used GLM-based approach [8], it is model-free and does not rely on the common assumptions of BOLD linearity or HRF uniformity. Secondly, unlike spatially multivariate approaches such as MVPA and RSA [10,46], this scheme treats entire runs, rather than blocks or trials, as its basic experimental unit. We demonstrated the feasibility of this approach in an individual subject, noting that further research is needed to quantify its strengths and limitations.

The last decade has seen a shift in cognitive neuroscience toward analysis methods that allow representation codes to vary between subjects [47]. This trend is most evident in the wide endorsement of multivariate pattern–information analyses, such as MVPA and RSA. These analyses exploit the high spatial resolution of fMRI and, instead of averaging the signal across voxels, rely on the consistency of spatial activation patterns within categories (MVPA) or across particular tokens (RSA). This way, within-subject spatial consistency can be propagated to population inference, even when fine-scale anatomy or functional organization differs between subjects (known as “the representational dissimilarity trick”; [48]).

While these multivariate analyses allow for variation in spatial organization, they are less tolerant to variations in the *temporal* dynamics of the signal. This is reflected in the model-based extraction of one statistic per voxel, either explicitly (when extracting betas from a GLM) or implicitly (when choosing a representative time point). In contrast, the proposed analysis scheme uses the consistency of the voxel’s time series across conditions as a measure of its sensitivity to the experimental manipulation. Thus, instead of applying the representational dissimilarity trick spatially, as in the commonly used MVPA, here, we apply it to the temporal dimension. This way, our approach (as well as other temporal correlation-based methods; see Table 1) is more resilient to model-resistant activation patterns that may characterize particular brain regions, populations, or subjects.

Our approach is also run-related rather than event- or block-related. With the exception of adaptation designs, in which the temporal dependence between events serves as the dependent measure (e.g., [49]), model-based approaches to fMRI analysis most often treat experimental events as temporally independent. This way, interaction between subsequent events is averaged out by trial randomization instead of being incorporated into the statistical model. TCA, on the other hand, exploits serial interactions as a source of meaningful information. As the timing and order of events are preserved across runs, any consistent temporal interaction at the cognitive [50], neural [51], or vascular level [12] is taken as a possible source of information instead of noise.

### 4.1. Limitations

#### 4.1.1. Passive Experimental Procedures

TWISTER randomization relies on temporal synchronization between runs. Unlike traditional approaches to fMRI, in which the design matrix can be adapted based on subjects’ behaviour in the scanner (such as response times, decisions, reported confidence, or later memory recall of experimental events), post hoc adaptations are not possible in TCA, as the design matrix is replaced by the red and blue reference time series. This makes TWISTER less suitable for experimental paradigms in which the timing or type of events is not experimentally controlled (for example, paradigms that rely on subjects’ responses).

#### 4.1.2. Power

Being model-free, our TCA analysis is more statistically sensitive when model assumptions are not met, but it is no match for model-based approaches in terms of statistical power when assumptions do hold. As an illustration, in the widely used GLM, noisy empirical data are fit to idealized regressors that represent hypothetical, noise-free activation. In TCA, in contrast, both sides of the correlation (the seed and reference time series) are affected by measurement noise, increasing the effect of measurement noise on the precision and sensitivity of the inference and bringing down statistical power.

#### 4.1.3. Order Effects

The brain is a plastic organ, and the first exposure to an event is almost guaranteed to affect the neural responses to future repetitions of the same event ([52], p. 231). Consequently, asymmetries in the temporal consistency between runs may not only reflect true asymmetries in the representational space or random noise but also systematic differences in processing that stem from run order, such as novelty or habituation effects (generally termed *Groundhog effects*; [53]) or systematic differences in head motion or signal drift due to scanner noise. This can be accounted for at the population level (by assigning a different run order to each subject) and partly at the single-subject level (as done here by splitting each run into two or three sub-runs and randomizing their order, by pre-processing raw data to account for head motion within and between runs, and by applying temporal filtering to account for slow drifts in the overall signal). However, not unlike traditional approaches to fMRI analysis, one should always keep in mind possible acquisition-order artifacts.

#### 4.1.4. Discarding of Negative Correlations

TCA uses Pearson correlations as an index of similarity between time series, with 0 standing for no similarity and 1 for perfect similarity. Therefore, negative correlations are discarded to avoid significant effects being driven by systematically opposite patterns of responses in different experimental runs. Crucially, this step only makes our test more *conservative* in potentially ignoring effects that are partly driven by such negative correlations.

In general, correlations between time series tend to be positive, making the effect of this step relatively minor. Whenever strong negative correlations are observed (this can happen in a paradigm like ours, for example, if the execution of a right hand movement is always coupled with the active suppression of a left hand movement), setting negative correlations to zero may fail the Hotelling–Williams test by giving rise to an inconsistent correlation matrix (technically, one that is not positive and semi-definite). In such cases, researchers may opt to skip this step and use raw correlations or adopt a less sensitive statistical test for comparison of correlations, such as Fisher’s Z test [54].

## 5. Conclusions

This study provides a first demonstration of a new model-free fMRI approach to functional neuroimaging. We suggest that for some research questions, statistical inferences can be made on the time series as a single unit, based on its temporal consistency with other time series. Moreover, not relying on a generative model of the measured signal, this approach is robust to violations of model assumptions such as additivity, scaling, and shift invariance. Therefore, it may be especially beneficial for the study of certain populations (e.g., clinical populations or developmental studies), brain regions (e.g., subcortical structures or associative cortices), and pharmacological interventions for which these generic model assumptions are less likely to hold. Designing experiments that lend themselves to model-free analysis, as a complement to more statistically powerful model-based tools, would be an important step in mitigating the literature bias against model-resistant activations.

## Figures and Tables

**Table 1:** Three model-free applications in fMRI analysis that use temporal correlations between subjects, voxels, or condition to infer functionality. “Between” factors are marked in red. TWISTER (fourth row) examines correlations between time series of the same voxel within the same subject but across different experimental conditions.

	Subjects	Voxels	Conditions
Intrasubject reliability (Hasson et al., 2010 [33])	Within	Within	Within
Intersubject correlation (Hasson et al., 2004 [34])	Between	Within	Within
Functional connectivity (Biswal et al., 1995 [31])	Within	Between	Within
TWISTER	Within	Within	Between

## Data Availability

Pre-processed, anonymized data is publicly available (www.github.com/matanmazor/TWISTER).
